# Exploring the key target molecules of angiogenesis in diabetic cardiomyopathy based on bioinformatics analysis

**DOI:** 10.3389/fendo.2025.1561142

**Published:** 2025-04-17

**Authors:** Fengli Hu, Ruixue Guo, Yaxin Zhi, Haijuan Hu, Ting Tang, Pengfei Wang, Ling Xue

**Affiliations:** ^1^ Department of Cardiology, Second Hospital of Hebei Medical University, Shijiazhuang, China; ^2^ Department of Internal Medicine, Hebei Medical University, Shijiazhuang, China

**Keywords:** diabeticcardiomyopathy, angiogenesis, bioinformatics, therapeutictargets, EFNB2

## Abstract

**Backgrounds:**

Diabetic cardiomyopathy has a very high incidence and serious clinical consequences, making it an urgent clinical problem to be solved. Angiogenesis is a significant phenotype in the occurrence and development of diabetic cardiomyopathy, especially the damage to angiogenesis of cardiac microvessels, which is inextricably linked to the cardiac risk of diabetic patients. In the current basic and clinical research, there is still a lack of treatment methods that directly target the angiogenesis of diabetic cardiomyopathy. This study hopes to discover the key molecules related to diabetic cardiomyopathy and angiogenesis damage, to provide ideas for possible interventions.

**Methods:**

Sequencing data of animals and cells were obtained from the GEO database, and differentially expressed genes were analyzed. Subsequently, the angiogenesis-related genes were clustered for functional and pathway analysis. Then, the microangiogenesis of the diabetic mice and the angiogenesis changes of high glucose-stimulated HUVECs were verified, and the top three genes related to diabetic cardiomyopathy and angiogenesis were verified using western blot.

**Results:**

24 differentially expressed genes associated with angiogenesis were found in GSE241565(human) and GSE215979(mice). Among them, 11 genes showed the same trend in the two databases. Then CD31 staining of diabetic mice hearts showed that microvascular angiogenesis was impaired, high glucose-stimulated HUVECs decreased tube formation, and wound healing migration was weakened. Finally, the top 3 genes most associated with diabetic cardiomyopathy were verified, and there was no significant difference between the changes of Edn1 and Lepr. At the same time, Efnb2 was significantly increased under high glucose stimulation.

**Conclusion:**

Combined with the sequencing data of animal and cell models of diabetic cardiomyopathy, the differential genes associated with angiogenesis were screened. These findings not only elucidate a novel molecular axis linking angiogenesis damage to diabetic cardiomyopathy but also highlight Efnb2 as a potential therapeutic target.

## 1 Introduction

In 2019, 463 million adults were reported to have diabetes, and this number is expected to reach 700 million by 2045 (1). Cardiovascular disease is a common complication in diabetics and is thought to be the leading cause of death and disability in diabetic patients thus the continued surge in the prevalence of diabetes has increased the burden of diabetic cardiomyopathy (DCM) and has become a key issue in the diabetes field (2–4). Unlike other cardiovascular complications, DCM is usually absent in the absence of coronary artery disease or hypertension but is often associated with left ventricular diastolic dysfunction, ventricular hypertrophy, and myocardial fibrosis (3, 5). Currently, there are many mechanisms involved in the development of DCM, and many studies have focused on myocardial inflammation (6, 7), pyroptosis (7), ferroptosis (8–11), and so on. Whereas We are very interested in vascular injury, especially angiogenesis.

As we all know, the effect of diabetes on angiogenesis may not be consistent in different organs (12, 13). On the one hand, the increase in pathological angiogenesis is associated with diabetic retinopathy and nephropathy; On the other hand, in diabetes, angiogenesis in the coronary and peripheral arteries of the heart is weakened (14, 15). Studies have found that microvascular angiogenesis is affected in diabetes or metabolic disorders (16, 17). Endothelial cell damage is thought to be the origin of microvascular dysfunction in early type 2 diabetes (18), and as many researchers have assumed, impaired angiogenesis in diabetic cardiomyopathy is a key factor in the development of serious adverse cardiovascular events in many patients with diabetes (19). Angiogenesis is significantly affected in DCM, and key genes in DCM are enriched in the angiogenesis process (20), but there is still a lack of specific interventions targeting angiogenesis in DCM, and no key molecules affecting angiogenesis in DCM have been identified.

With the development of a variety of biological science and technology, many researchers are concerned about the molecular changes associated with diabetic cardiomyopathy. Identifying these specific molecules, can not only help to enhance the possibility of diagnosis and treatment (21–23) but also predict the risk of heart failure (24), which can help to identify high-risk patients. Thus based on the tissue and cell sequencing data of diabetic cardiomyopathy, combined with relevant databases, we screened out genes that are significantly associated with angiogenesis when diabetic cardiomyopathy occurs. Subsequently, similar animal and cell models were constructed to verify the differential gene expression, suggest the existence of key molecules, and provide the possibility for the next targeted intervention.

## 2 Materials and methods

### 2.1 Data preparation

By searching the NCBI GEO database(https://www.ncbi.nlm.nih.gov/geo) for diabetic cardiomyopathy and diabetes-related vascular injury, two datasets piqued our interest. Among them, GSE241565 (25) was experimented with human umbilical vein endothelial cells (HUVEC), and high-throughput sequencing was performed after 8 days of stimulation with different sugar concentrations (5.5 mM *vs*. 30 mM). Another dataset GSE215979 (26) contains information on hearts of STZ-induced diabetic mice. The list of genes associated with human angiogenesis was obtained from the GeneCards database (https://www.genecards.org/), while the list of genes associated with angiogenesis in MICE was downloaded from the Mouse Genome Informatics(https://www.informatics.jax.org/). Otherwise, genes associated with human diabetic cardiomyopathy were obtained from the Comparative Toxicogenomics Database(CTD, https://ctdbase.org/).

### 2.2 Differential expressed genes screening

GSE241565 differentially expressed genes were analyzed by online GEO2R, in which all genes of Padj<0.05 were included in the preliminary analysis set. P value adjustment is calculated by Benjamini & Hochberg (False discovery rate), and online analysis is based on R 4.2.2 and limma 3.54.0. In GSE215979, we performed differentially expressed gene analysis using the R package “limma”, and P<0.05 was set as the identification threshold. In order not to miss potentially differentially expressed genes, we did not limit fold change values. Subsequently, genes associated with angiogenesis in the two datasets were merged by homologous gene conversion of different species. Heatmap was plotted by https://www.bioinformatics.com.cn (last accessed on 10 Nov 2023), an online platform for data analysis and visualization.

### 2.3 Protein-protein interaction and functional enrichment analysis

The selected differentially expressed genes were analyzed using the STRING database(https://cn.string-db.org/) for protein-protein interaction network analysis and interaction network diagram. In the next step, the functional and pathway enrichment analysis of differentially expressed genes was performed by DAVID Bioinformatics (https://davidbioinformatics.nih.gov/). The visualization of the pathway analysis was performed using the WeBio website (27). Among them, the most relevant molecular pathway analysis of the KEGG pathway was performed using KEGG online analysis (https://www.genome.jp/kegg/). And the subcellular localization and the membrane type of proteins were predicted in DeepLoc-2.1 (https://services.healthtech.dtu.dk/services/DeepLoc-2.1/).

### 2.4 Animal model

All C57BL/4N mice (male, 7 weeks, 21–25g) were purchased from Vital River and randomly divided into diabetes mellitus group(DM) and negative control group(NC) after adaptive feeding. All mice live in a quiet environment, with 12 hours of alternating light and dark and regular disinfection and sterilization. Mice have free access to food and clean drinking water. Diabetic model mice are fed with a High-fat diet (HFD) and injected intraperitoneally with STZ (30 mg/kg) dissolved in sodium citrate buffer(pH4.5, sterile, Solaibio, C1013). The control mice were intraperitoneally injected with sodium citrate buffer solution at the same time and in the same body weight ratio.

### 2.5 Immunofluorescence staining

For immunofluorescence staining of cardiac tissue, OCT-embedded fresh cardiac tissue is sectioned using a cryostat (thickness of 5 microns), and the tissue sections are spread flat on a glass slide. Equilibrate for 10 minutes at room temperature followed by 3 washes in PBS, followed by blocking with 5% goat serum for 30 minutes at room temperature, followed by CD31 (1:50, mouse primary antibody from GeneTex. GTX20218) at 4°C overnight. On the second day, the fluorescent secondary antibody (1:100) was incubated for 1 hour at room temperature in the dark, and the mounting was performed with DAPI containing quenching inhibitor after washing. Finally, the images were observed and photographed under a confocal microscope (Olympus).

### 2.6 Cell culture

Human Umbilical Vein Endothelial Cells(HUVECs) were routinely cultured with Dulbecco’s modified Eagle medium (DMEM) (containing 25 mM glucose) containing 10% fetal bovine serum and 1% penicillin/streptomycin, 30 mM glucose were used as high-glucose stimulation conditions, and the cells were collected for follow-up experiments after 48 hours of normal culture and high-glucose stimulation. The incubator is set at 37°C, 5% carbon dioxide, and 95% humidity.

### 2.7 Wound healing assay

To test the angiogenesis ability of endothelial cells, a wound-healing assay was performed. HUVECs were planted in 6-well plates. After LG or HG stimulation for 48 hours, a 200-μl pipette tip was used to vertically and straightly scratch. Then, sterile PBS was gently rinsed 3 times to remove the cell debris produced by the scratch. Finally, the DMEM medium without FBS was changed to continue the culture, and pictures were taken under a light microscope at 0h, 12h, and 24h. After collecting the pictures, the scratch migration length was measured by ImageJ analysis software, and the scratch in the same well was measured at least 3 times, and the average value was taken to calculate the migration rate.

### 2.8 Tube formation assay

Matrigel (Corning, USA) at 4°C overnight in advance, and pre-cool the 24-well plate and pipette tip in the refrigerator. The next day, evenly apply the melted matrix gel to the 24-well plate (20 μl per well) and then incubate in a cell culture incubator for 30 minutes. Digest and resuspend HUVECs for 72 hours, inoculate 15×104 per well on the coated plate, continue to culture with DMEM medium without FBS, and observe the tube formation phenomenon with an inverted optical microscope and take pictures. Finally, use the Image J software plug-in “Angiogenesis Analyzer” for quantitative analysis of tube formation.

### 2.9 Western blot

HUVECs were lysed for 30 minutes to one hour using RIPA lysis buffer containing 1% PMSF, and the supernatant was collected after centrifugation at 12,000 rpm for 10 minutes at 4°C. The protein was then quantified using a BCA protein assay kit (Solaibio, China). After determining the appropriate concentration, 5× loading buffer was added and the sample was boiled at 95-100°C for 5-10 minutes. Proteins were separated by protein electrophoresis using 10% SDS-PAGE gel and then transferred to a PVDF membrane (Millipore, Burlington, MA, USA). The membrane was blocked with 5% milk at room temperature for one hour and then incubated with the primary antibodies against EDN1 (1:500, Wanleibio, WL02780, China), LEPR (1:1000, Wanleibio, WL0162a China), EFNB2 (1:1000, Abways, CY7104, China) and GAPDH (1:1000, Huabio, R1210-1, China) at 4°C overnight. The next day, the HRP secondary antibody was incubated at room temperature for 1 hour and then developed using a bio-rad gel imager. GAPDH was used as an internal reference protein for standardization and quantitative analysis was performed using ImageJ software.

### 2.10 Statistical analysis

All data were presented as mean ± standard error of the mean (SEM), and the statistical analysis was conducted by GraphPad Prism 8.0 (GraphPad Software, Inc., San Diego, CA, USA). Statistical differences between the two groups were analyzed using an unpaired t-test. In addition, P<0.05 was considered statistically significant.

## 3 Results

### 3.1 Screening of differential DCM genes associated with angiogenesis in human-derived sequencing information

**Figure 1:**
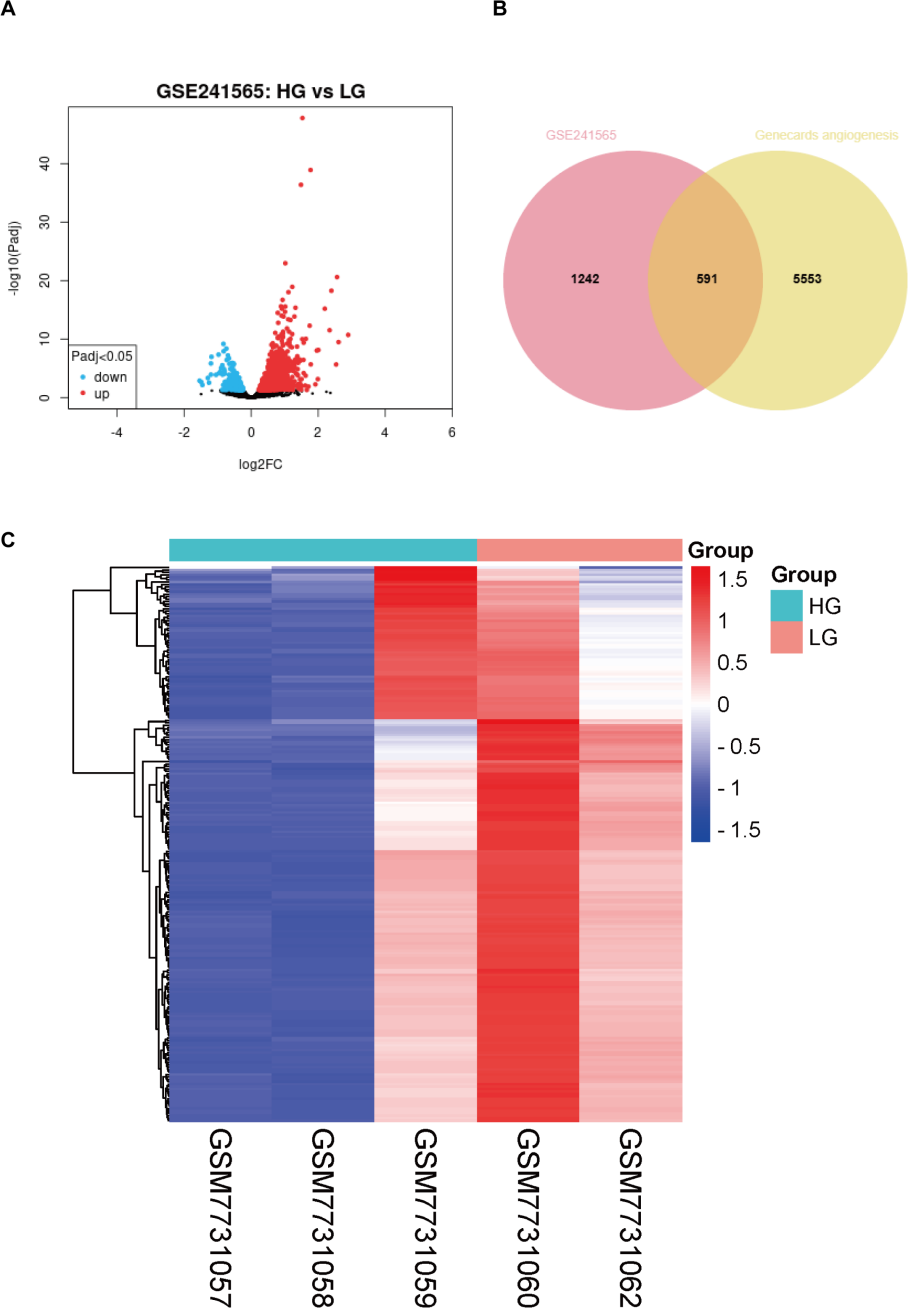
Analysis of differentially expressed genes associated with angiogenesis in high-glucose-stimulated HUVECs. **(A)** Volcano plot of differentially expressed genes from the GSE241565 dataset. **(B)** Venn diagram of GSE241565 differentially expressed genes and angiogenesis related genes in Genecards. **(C)** Heat map of differentially expressed genes associated with angiogenesis in HG-stimulated HUVECs. LG, low glucose; HG, high glucose; HUVECs, Human umbilical vein endothelial cells.

First, we performed a differentially expressed gene analysis on the GSE241565 dataset using an online GEO2R analysis tool. The data available for analysis in this dataset include sequencing data for HUVECs stimulated by HGs: GSEM7731057, GSEM7731058, and GSEM7731059. The LG group as a control group contains 2 samples: GSEM7731060, and GSEM7731062 (where the GSEM7731061 dataset does not upload raw data). After analysis, it was found that there were 1833 differential genes between groups, including 462 up-regulated genes and 1371 down-regulated genes (**Figure 1A**). Subsequently, we searched the human gene database (Genecards) for all genes associated with “angiogenesis”, for a total of 6144. Finally, 591 differentially expressed genes related to human angiogenesis were obtained (**Figure 1B**). Then, these 591 genes were heatmapped (**Figure 1C**), and the exact differences between groups were found.

### 3.2 Screening of differential DCM genes associated with angiogenesis in mouse-derived sequencing information

**Figure 2:**
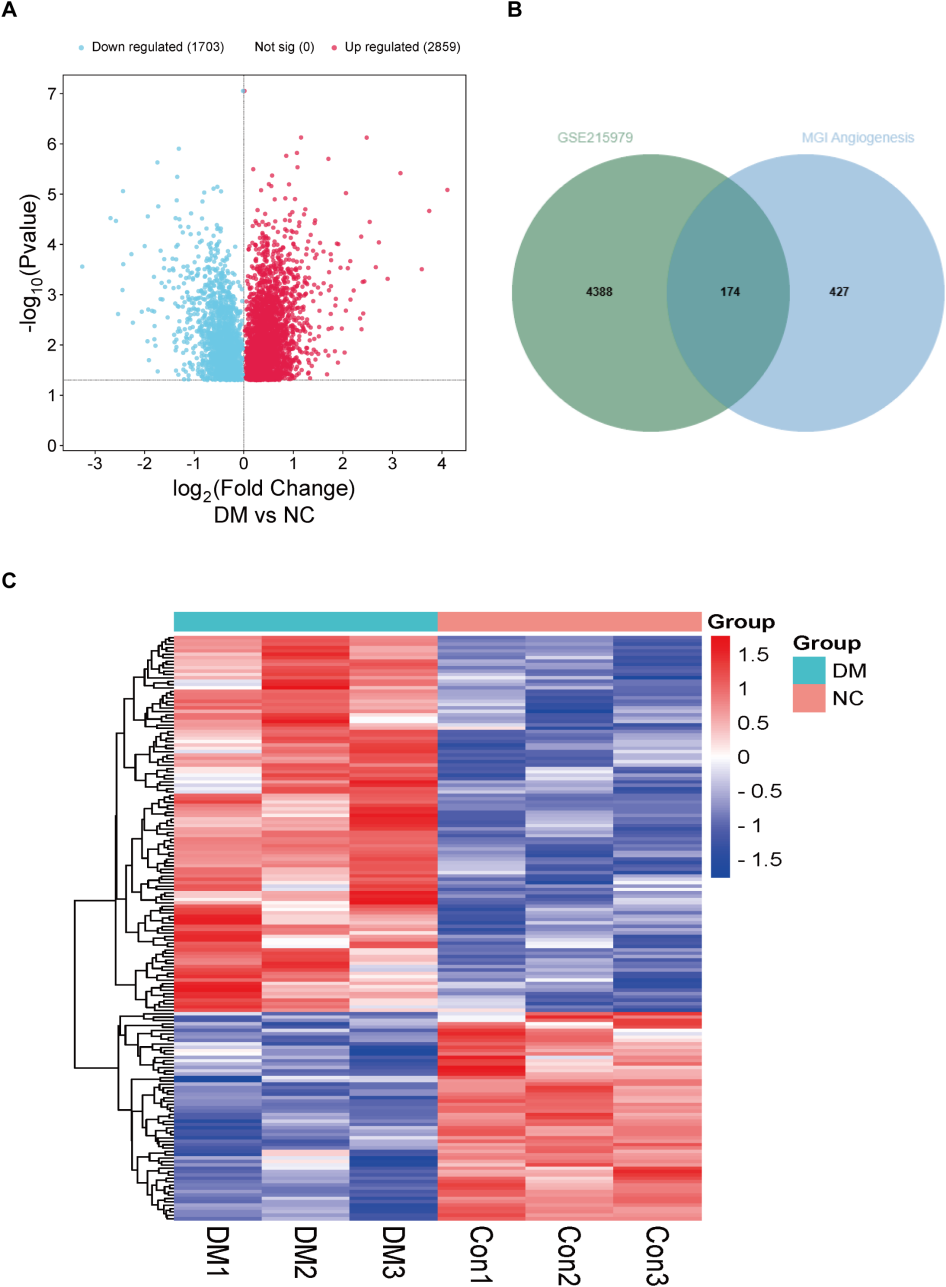
Analysis of differentially expressed genes associated with angiogenesis in hearts of DM mice. **(A)** Volcano plot of differentially expressed genes from the GSE215979 dataset. **(B)** Venn diagram of GSE215979 differentially expressed genes and angiogenesis related genes in MGI. **(C)** Heat map of differentially expressed genes associated with angiogenesis in DM mice.NC, negative control; DM, diabetes mellitus.

For differential gene screening in animal models of DCM, we performed a “limma” differential analysis of GSE215979, which included sequencing results of diabetic and negative control mouse hearts with 3 samples each. As a result, 4562 differential genes were obtained, of which 2859 were up-regulated and 1703 were down-regulated (**Figure 2A**). Next, 601 genes associated with mouse angiogenesis were extracted from the mouse genome informatics database (MGI). Similarly to the previous one, it was found that there were 174 differential genes for DCM associated with angiogenesis in mice (**Figure 2B**). These differential genes were also heat-mapped to visualize the differences between groups (**Figure 2C**).

### 3.3 Analysis of gene interactions associated with DCM angiogenesis

**Figure 3:**
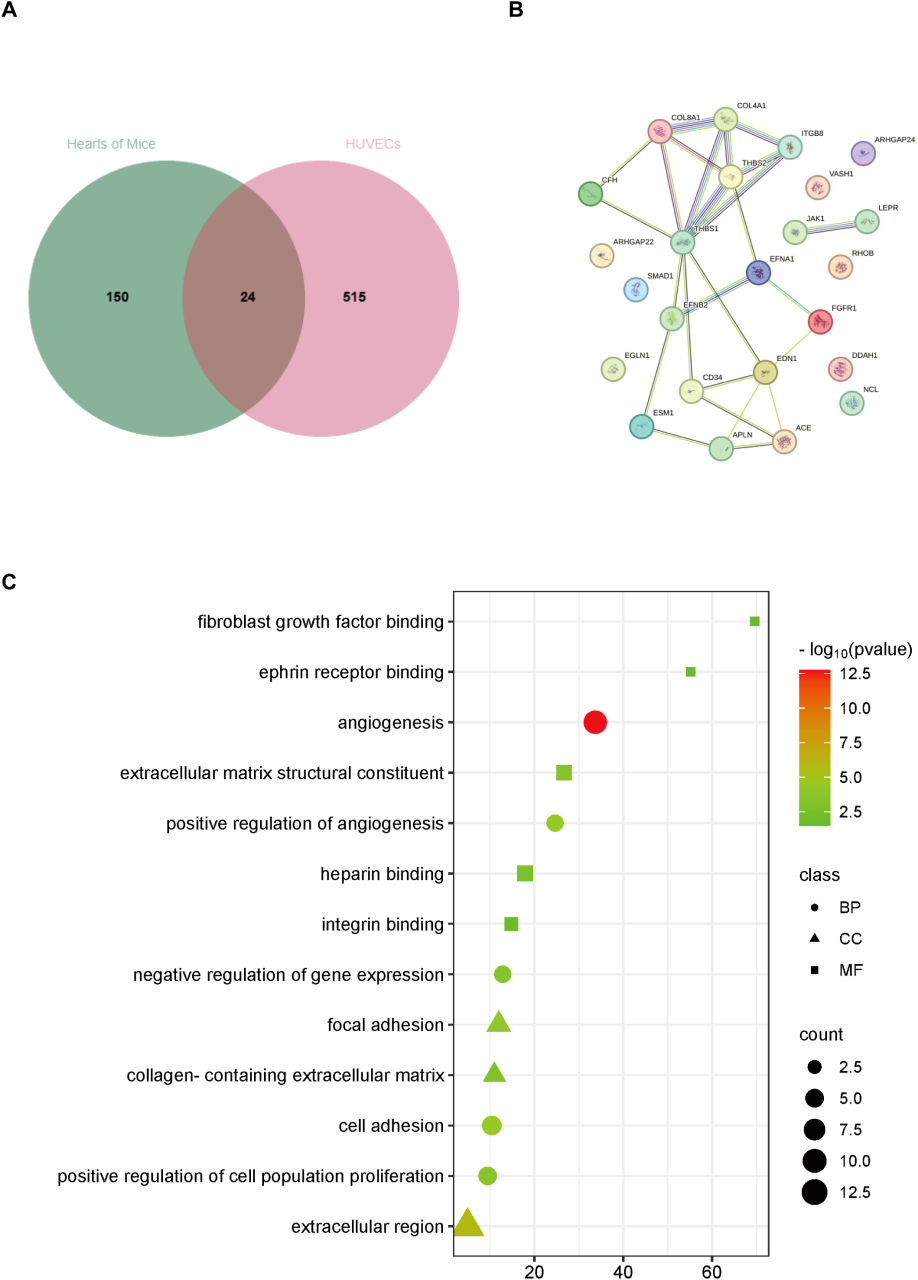
Analysis of differentially expressed genes associated with angiogenesis both in hearts of DM mice and HG-stimulated HUVECs. **(A)** Venn diagram of differentially expressed genes. **(B)** Protein-protein interaction network analysis of differentially expressed genes. **(C)** GO analysis of differentially expressed genes. BP, biological process; CC, cellular component; MF, molecular function.

Considering that the genes of humans and mice do not correspond completely, we homologous converted 591 differential genes related to angiogenesis in HUVECs to obtain 539 corresponding mouse genes. Subsequently, it was integrated with 174 differential genes related to angiogenesis in mice, and finally, 24 angiogenesis-related genes were obtained that were differentiated in both humans and mice (**Figure 3A**). To clarify the correlation between these differential genes, we mapped the protein-protein interactions using the STRING online analysis tool (**Figure 3B**). We not only focus on the interactions between differential genes but also explore pathways that are significantly associated with angiogenesis impairment in DCM. Next, GO(Gene Ontology) analysis found that all differential genes involved BP(biological process) included angiogenesis, cell adhesion, positive regulation of angiogenesis, positive regulation of cell population proliferation, negative regulation of gene expression, CC(cellular component) included extracellular region, focal adhesion, collagen-containing extracellular matrix, and finally MF(molecular function) included extracellular matrix structural, constituent, heparin-binding, integrin binding, fibroblast growth factor binding, ephrin receptor binding (**Figure 3C**).

### 3.4 Analysis of gene pathway enrichment associated with DCM angiogenesis

**Figure 4:**
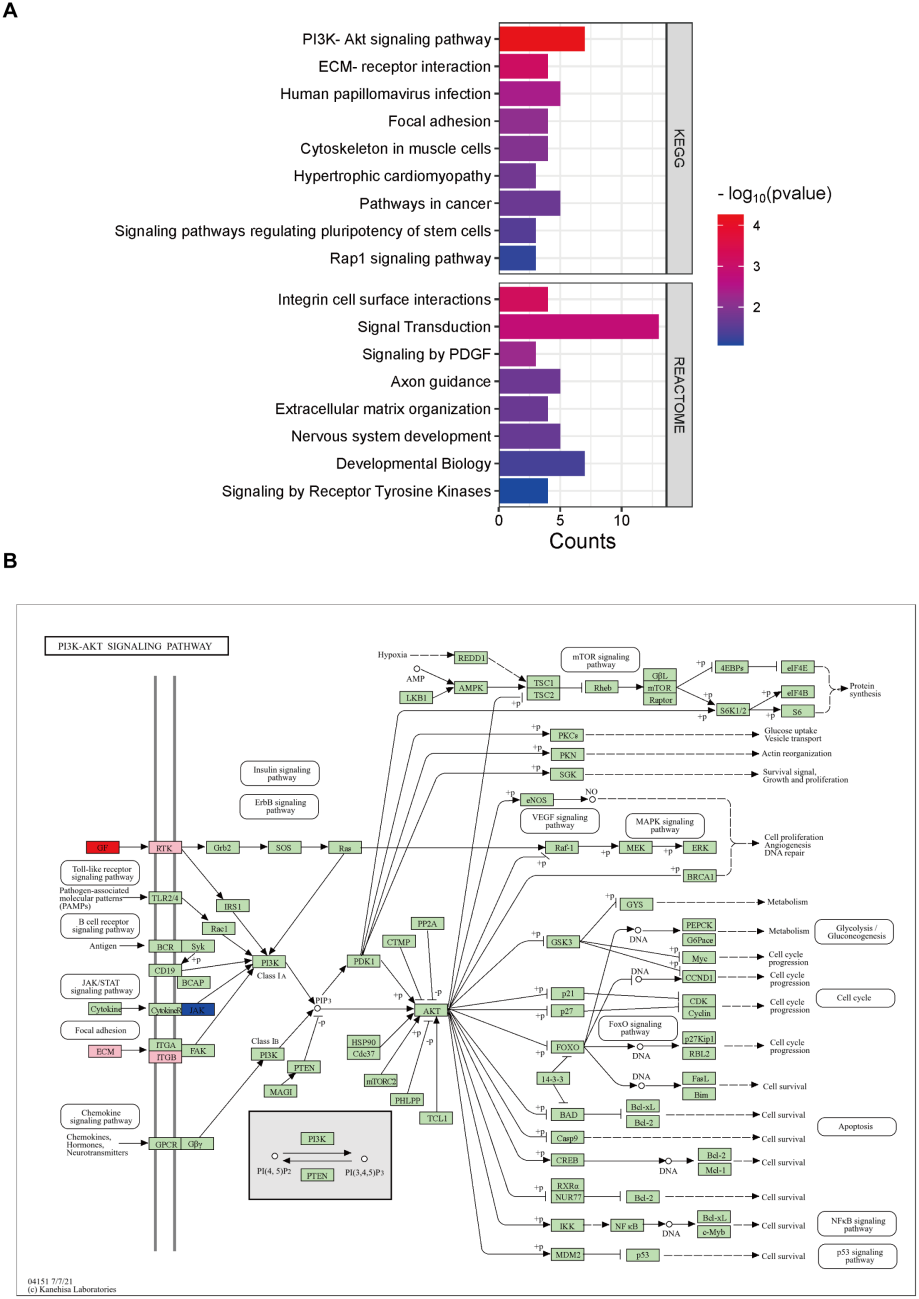
Analysis of relevant pathways about differentially expressed genes associated with angiogenesis. **(A)** KEGG and REACTOME analysis of differentially expressed genes. **(B)** KEGG pathway analysis of PI3K-AKT signaling pathway. Pink is chosen as the background color, where the up-regulated genes are labeled red and down-regulated are blue.

Pathway enrichment analysis was performed for angiogenesis-related differential genes, including KEGG and REACTOME pathway analysis, respectively (**Figure 4A**). It is associated with PI3K-Akt signaling pathway, Human papillomavirus infection, Pathways in cancer, ECM-receptor interaction, Focal adhesion, Cytoskeleton in muscle cells, Hypertrophic cardiomyopathy, Signaling pathways regulating pluripotency of stem cells, Rap1 signaling pathway in the KEGG pathway. And in the REACTOME pathway, includes Signal Transduction, Developmental Biology, Axon guidance, Nervous system development, Integrin cell surface interactions, Extracellular matrix organization, Signaling by Receptor Tyrosine Kinases, and Signaling by PDGF.

Of all the KEGG pathways involved, the PI3K(Phosphatidylinositol 3-Kinase)-AKT signaling pathway is the first and significantly related pathway. We then performed a further pathway map analysis, in which the up-regulated genes were labeled in red, down-regulated in blue, and the background color was pink by default (**Figure 4B**). The results showed that GF(Growth Factor), RTK(receptor tyrosine kinase), ECM(Extracellular Matrix), ITGB(Integrin Beta), and JAK(Janus Kinase) in the upstream of the signaling pathway were associated with impaired angiogenesis in DCM, in which GF was up-regulated and JAK was down-regulated.

### 3.5 Verification of angiogenesis in diabetic cardiomyopathy

**Figure 5:**
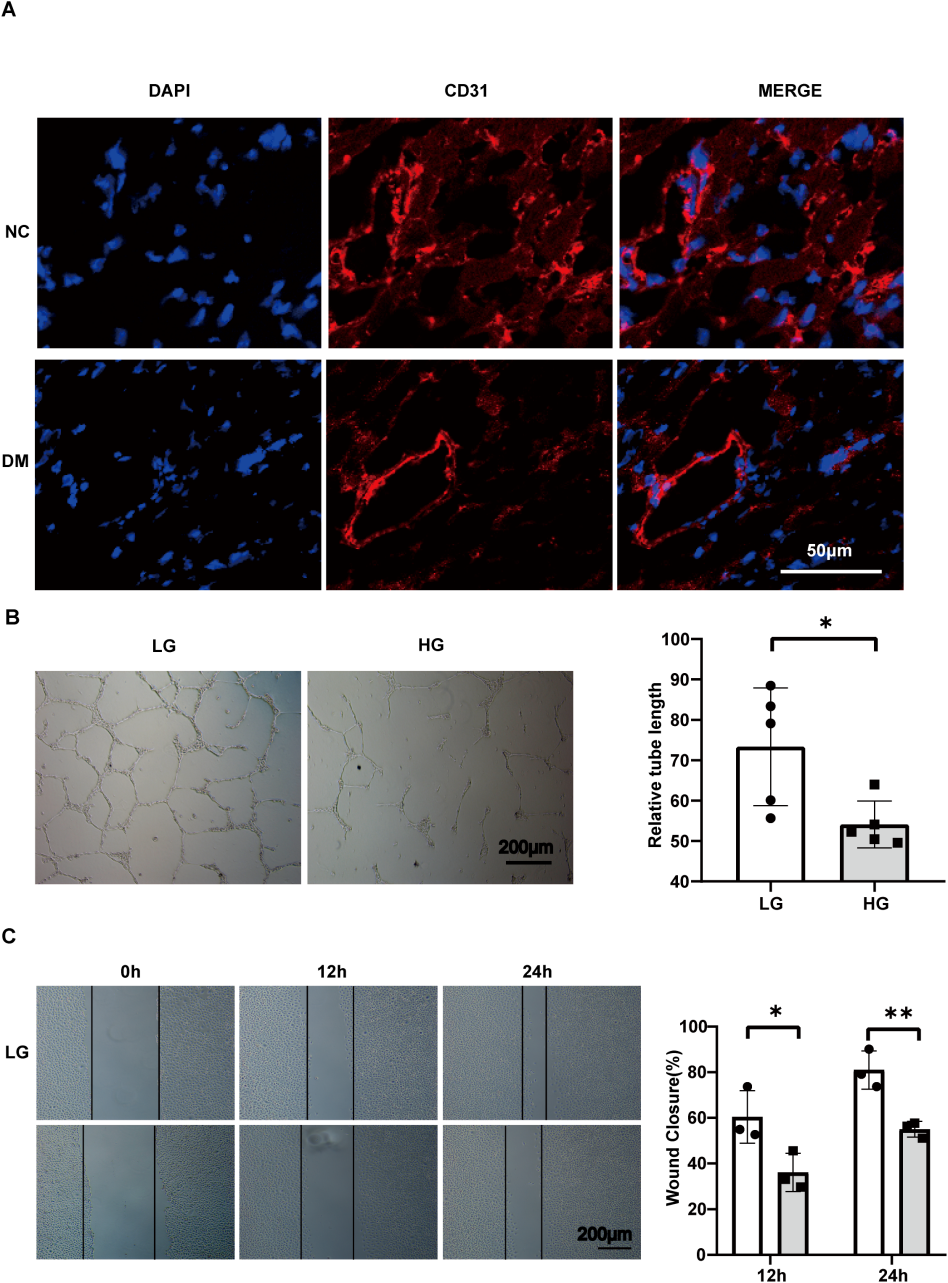
Angiogenesis impairment occurs in the hearts of DM mice and in HG-stimulated HUVECs. **(A)** Confocal image of cardiac microangiogenesis (CD31, red) in mouse heart tissue, scale bar, 50 μm. **(B)** Tube formation assay and related analysis of HUVECs, scale bar, 200 μm. n=5. **(C)** Wound healing assay and related analysis of HUVECs, scale bar, 200 μm. n=3. Data represent the mean ± SEM. *P<0.05, **P<0.01. NC, negative control; DM, diabetes mellitus; LG, low glucose; HG, high glucose; HUVECs, Human umbilical vein endothelial cells.

To assess cardiac angiogenesis in diabetic cardiomyopathy mice, we performed immunofluorescence staining on frozen sections of heart tissue (**Figure 5A**). Among them, CD31(red) represents an indicator of angiogenesis, and the CD31 staining of microvessels in the heart of DM mice is attenuated overall. Subsequently, HG was used in cells as an adverse stimulus in diabetes mellitus, and angiogenesis-related experiments were evaluated. Among them, the tube formation function of HUVECs is weakened at HG, and the associated tube length is significantly reduced (*P*= 0.0253) (**Figure 5B**). In addition, the results of the scratch-healing assay showed that high glucose stimulation impaired the migration ability of HUVECs, and the mobility rate was significantly reduced at 12 h and 24 h (**Figure 5C**). There was a significant difference between 12 and 24 hours(*P* = 0.041720, 0.007644 respectively).

### 3.6 Western blotting were carried out to verify differentially expressed genes

**Figure 6:**
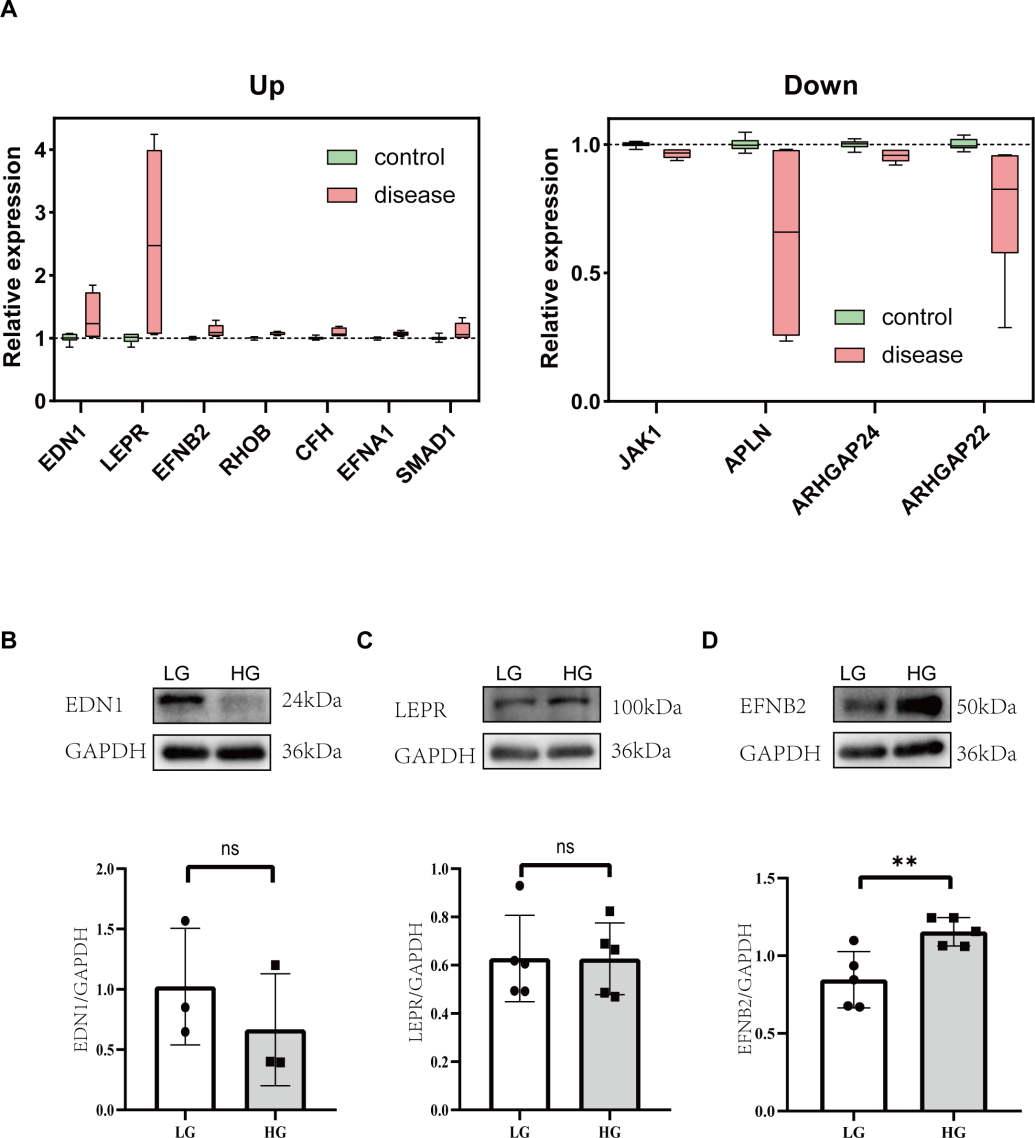
Analysis of proteins associated with impaired angiogenesis in diabetic cardiomyopathy. **(A)** Box plots of the relative changes in differentially expressed genes with consistent trends. **(B)** Representative western blot images and relative analysis of EDN1 on HG-stimulated HUVECs. n=3. **(C)** Representative western blot images and relative analysis of LEPR on HG-stimulated HUVECs. n=5. **(D)** Representative western blot images and relative analysis of EFNB2 on HG-stimulated HUVECs. n=5. Data represent the mean ± SEM. *P<0.05, **P<0.01.LG, low glucose; HG, high glucose; HUVECs, Human umbilical vein endothelial cells; EDN1, endothelin 1; LEPR, leptin receptor; EFNB2, ephrin B2.

Integrating all sequencing data, there are 11 genes with the same change trend: EDN1, LEPR, EFNB2, JAK1, RHOB, CFH, APLN, EFNA1, SMAD1, ARHGAP24, ARHGAP22. Among them, 7(EDN1, LEPR, EFNB2, RHOB, CFH, EFNA1, SMAD1) were upregulated and 4(Jak1, Apln, Arhgap24, Arhgap22) were downregulated. To gain a better understanding of the genes involved, the subcellular localization and the membrane type of proteins were predicted in DeepLoc-2.1 (**Table 1**). These genes were then searched in the CTD (Comparative Toxicogenomics Database) for genes/proteins related to diabetic cardiomyopathy, and reference scores for all genes were obtained (**Table 1**). Firstly, the expression of each gene was standardized in the control group, and a box plot was drawn (**Figure 6A**). Finally, the top 3 proteins with the CTD reference scores were evaluated by western blotting. There was no significant difference between the groups in EDN1 and LEPR (*P* = 0.4082, 0.9896 respectively) (**Figures 6B, C**), while EFNB2 was significantly increased when stimulated by high glucose (*P* = 0.0094) (**Figure 6D**).

**Table 1:** Protein localization and membrane type analysis corresponding to differentially expressed genes with consistent trends.

Gene	Protein	CTD Inference Score	Protein_ID	Localizations	Membrane types
EDN1	endothelin 1	40.2	AAA52339.1	Extracellular	Soluble
LEPR	leptin receptor	24.85	AAB09673.1	Cell membrane	Transmembrane
EFNB2	ephrin B2	22.77	URS64290.1	Extracellular	Transmembrane|Soluble
JAK1	Janus kinase 1	20.31	BAE02826.1	Cytoplasm Cell membrane	Peripheral|Soluble
RHOB	ras homolog family member B	19.72	KAI4033686.1	Cell membrane	Lipid anchor
CFH	complement factor H	17.31	CAA30403.1	Extracellular	Soluble
APLN	apelin	14.14	AAF25815.1	Extracellular	Soluble
EFNA1	ephrin A1	12.48	AAH95432.1	Cell membrane	Lipid anchor
SMAD1	SMAD family member 1	9.76	KAI4027178.1	Nucleus	Soluble
ARHGAP24	Rho GTPase activating protein 24	6.38	KAI4026049.1	Cytoplasm	Peripheral|Soluble
ARHGAP22	Rho GTPase activating protein 22	6.31	KAI4075927.1	Cytoplasm	Peripheral|Soluble

## 4 Discussion

Diabetic cardiomyopathy increases significantly with the rapid increase in the prevalence of diabetes, and the pathological processes involved are very complex, including diastolic dysfunction, myocardial fibrosis, and microvascular dysfunction. As many researchers hypothesize, impaired microvascular angiogenesis in diabetic cardiomyopathy may be the culprit behind the occurrence of serious adverse cardiovascular events in many patients with diabetes. Although many drugs and biological therapies are currently dedicated to improving diabetic cardiomyopathy-related lesions, there is still a lack of interventions targeting diabetic cardiomyopathy angiogenesis. We hope to combine sequencing databases and experimental studies to screen for differential molecules and signaling pathways associated with angiogenesis in diabetic cardiomyopathy, thereby providing the possibility of specific targeted interventions in the future.

Our transcriptomic analysis revealed significant enrichment of differentially expressed genes in the PI3K-AKT signaling pathway, and the PI3K-AKT pathway is an important intracellular signal transduction pathway, which is involved in the regulation of cell survival, proliferation, metabolism, and apoptosis. Emerging evidence further highlights the central role of PI3K-AKT signaling as a regulatory hub for diverse interventions in DCM (28–30). For instance, NLRP3 inflammasome knockout in endothelial progenitor cells (EPCs) was reported to rescue angiogenesis in diabetic mice with myocardial infarction, suggesting that the PI3K/AKT/mTOR cascade acts as a downstream effector of NLRP3-mediated inflammatory responses (31). Additionally, innovative approaches such as ultrasound-mediated microbubble cavitation have demonstrated dose-dependent pro-angiogenic effects in diabetic models, mechanistically attributed to PI3K-AKT-eNOS pathway activation and subsequent improvement in cardiac function (32). These data collectively position PI3K-AKT signaling as a critical mediator of both metabolic adaptation and vascular remodeling in diabetic hearts.

The differences between EDN1 and LEPR in our experimental validation did not show a statistical difference, but previous studies have identified EDN1 as a potential biomarker for DCM (33, 34). A DCM dataset (GSE62203) has been used to find that PGK1, LDHA, and EDN1 may promote M1 macrophage polarization in DCM (33). LEPR, on the other hand, is a leptin receptor that has been found to play an important role in cardiac hypertrophy, heart failure, and diabetes mellitus (35, 36). Therefore, these molecules may have different changes at different stages of DCM, and further research is needed to clarify their specific roles.

Further We found a significant increase in EFNB2 (EphrinB2) expression in HG-stimulated HUVECs, consistent with sequencing data. Previous studies have demonstrated that EphrinB2 plays a key role in angiogenesis and lymphangiogenesis (37, 38). In heart-related studies, researchers have high hopes for its ability to promote angiogenesis (39), but some studies have found that it plays a profibrotic role in cardiac fibrosis (40). However, recent studies have found that EphrinB2 prevents ischemic cardiac remodeling and dysfunction after myocardial infarction by activating the cardiac lymphatic vessels to produce signaling pathways (41). In addition, diabetes has been found to increase the expression of Ephrin-B2 in cerebral blood vessels and pericytes and increase cerebral neovascularization, which may be related to impaired cognitive function in diabetic patients (42). EphrinB2 elevation in diabetic cardiomyopathy may be a compensatory protective mechanism, or it may also play a multiple role in angiogenesis. More studies are still needed to uncover the role of EphrinB2 in angiogenesis in diabetic cardiomyopathy. However, screening of sequencing data can quickly and cost-effectively focus on key possible targets in many regulatory molecules.

Similar to relative studies, we also found that angiogenesis is suppressed in diabetic cardiomyopathy. Some studies have found that interventions such as apelin (17, 43), adipsin (44), cilostazol (45), and salvianolic acid B (46) can help improve angiogenesis in DCM. In addition, genetically engineered cell therapies (47) and stem cell transplantation (48) have also shown therapeutic potential. The emerging intervention regimens have shown certain therapeutic potential, but more efforts are still needed for targeted interventions based on key molecules and further clinical translation.

But inevitably, this study also has some limitations. First, we identified 11 genes associated with angiogenesis, but only the first 3 were immunoblotted. It is possible that other molecules also played an important role, but further validation is still needed. Second, we focused on angiogenesis in diabetic cardiomyopathy but did not link clinical information, and included patients with diabetic cardiomyopathy for clinical sample validation. Finally, focusing only on molecular changes cannot fully and accurately explain the changes in angiogenesis, and more experiments are still needed to discover the related regulatory pathways and regulatory patterns. These limitations require more time and experimental validation and are the next step we need to consider for further study.

## 5 Conclusion

In summary, this study systematically investigated angiogenesis impairment in DCM through integrated bioinformatics analysis, preclinical models, and molecular validation. By combining transcriptomic profiling of human and murine datasets with functional experiments in diabetic mice and high glucose-stimulated endothelial cells, we identified Efnb2 as a central regulator of microvascular dysfunction in DCM. These findings not only elucidate a novel molecular axis linking angiogenesis damage to DCM but also highlight Efnb2 as a potential therapeutic target.

## Data Availability

The original contributions presented in the study are included in the article material. Further inquiries can be directed to the corresponding author.
